# Automatic Tuning and Matching for NMR Probes Based on Physics-Informed Conditional Neural Processes

**DOI:** 10.3390/s26123724

**Published:** 2026-06-11

**Authors:** Zhida Zhai, Zhenggang Li, Ying He, Yaohong Wang, Chenjun Zhu, Weifeng Wu, Yitong Lin, Huijun Sun

**Affiliations:** 1 Fujian Provincial Key Laboratory of Plasma and Magnetic Resonance, Department of Electronic Science, Xiamen University, Xiamen 361005, China; 33320231150367@stu.xmu.edu.cn (Z.Z.); lizhenggang@stu.xmu.edu.cn (Z.L.); heyingpo3a@163.com (Y.H.); 33320231150382@stu.xmu.edu.cn (Y.W.); 33320241150412@stu.xmu.edu.cn (C.Z.); 36620241150498@stu.xmu.edu.cn (W.W.); linyitong1@stu.xmu.edu.cn (Y.L.); 2 Q.One Instruments Ltd., Wuhan 430075, China

**Keywords:** NMR probe, automatic tuning and matching, conditional neural processes, few-shot learning

## Abstract

The NMR resonator is the sensor responsible for transmitting RF pulses and receiving detection signals, and its tuning and matching are crucial to acquiring high-sensitivity NMR signals. Automated tuning and matching (ATM) is therefore essential for rapid, accurate, and continuously efficient testing. Existing NMR ATM methods still primarily rely on iterative search strategies, whose dominant cost arises from repeated hardware measurements and waiting periods, often requiring multiple measurement cycles before convergence. The emergence of in situ NMR detection of high-concentration ionic samples has further increased the demand for real-time, rapid ATM with a large dynamic range, posing a major challenge to conventional approaches. This paper proposes a physics-informed few-shot learning method for automatic tuning and matching over wideband and multi-resonance-frequency NMR scenarios. The tuning-and-matching problem is formulated as a structure and frequency-conditioned function regression task, and a conditional neural process (CNP) is introduced to learn cross-task priors and directly predict the states of tunable components from only a small number of real-machine context measurements. A physics regularizer based on the local sensitivity of the input impedance is further designed to impose stronger penalties on errors under high-Q narrowband operating conditions without relying on proprietary analytical circuit models. Simulation studies and real NMR experiments are conducted on multiple circuit topologies and multiple target frequencies using only a small number of NMR samples. The results demonstrate consistent improvements in key metrics, including accuracy of tuning and matching and the number of collected real-machine samples required per task. In particular, with only 100 sampled tuning/matching capacitor points and 20 on-hardware collected samples, the proposed method already delivers satisfactory tuning-and-matching performance. The method achieves an attractive accuracy–cost tradeoff across both cross-topology and cross-frequency scenarios, and shows strong potential for few-shot, rapid, real-time detection.

## 1. Introduction

Nuclear magnetic resonance (NMR) technology is based on the interaction of nuclear spins in the sample to be tested with an externally applied static magnetic field and RF field. It can non-destructively obtain molecular structural and dynamical information from within matter, and has become a fundamental tool in chemistry, biology, and medicine [1,2,3]. In NMR systems, the NMR resonator is the core component for excitation and reception of nuclear magnetic signals, and its performance directly determines the sensitivity of the system. Because magnetic resonance signals are extremely weak, high-Q L-C structures or microstrip transmission-line structures are typically employed to minimize transmission losses along the signal path [4]. Impedance matching at the operating frequency is the primary requirement of the NMR resonator. Impedance mismatch causes the operating frequency to deviate from the Larmor frequency and reduces the quality factor Q, which not only decreases RF power-transfer efficiency to the coil during transmission but also degrades the pickup of weak NMR signals, thereby substantially reducing the received signal-to-noise ratio [5]. In high-field NMR applications, the electrical performance of NMR resonators is highly sensitive to loading effects. When samples with different concentrations, shapes, or dielectric properties, such as chemical solutions with different salinities or biological tissues, are placed inside the NMR resonator, the filling condition inside the coil changes, which in turn shifts the input impedance and causes mismatch [6]. By readjusting the impedance-matching network to retune the operating frequency to the Larmor frequency and transforming the input impedance to the matching point, the adverse effects of the loading effect can be effectively mitigated, thereby improving the efficiency of RF excitation and SNR of the signal.

In the past, the tuning and matching of NMR probes relied on manual adjustment, which was inefficient and heavily dependent on the experience of operators. With the increasing demand for high-throughput experiments and automated testing, rapid and accurate automatic tuning and matching has become an indispensable function of modern NMR instruments. Existing research on automatic tuning and matching for NMR probes has focused primarily on iterative search methods based on the input reflection coefficients and their variants. A representative example is the ArduiTaM system proposed by Jouda et al. [7], which performs frequency sweeps around the Larmor frequency, monitors the dip position of the reflection curve, and iteratively adjusts the tuning and matching capacitors to achieve automatic tuning and matching across different channel bands. Sohn et al. proposed an improved iterative auto-search tuning algorithm [8] that first adjusts the tuning capacitor to pull the resonant frequency back to the Larmor frequency and then finely tunes the matching capacitor so that the input impedance is transformed to 50 Ω, thereby moving the impedance point to the center of the Smith chart. Although such search-based methods avoid explicitly modeling the complex structure of NMR resonators, the coupling among NMR resonators, and dielectric variations induced by different samples, their essence remains a closed-loop iterative search on physical hardware. They typically require repeated measurements and iterative adjustments to approach the optimal matching point. Each search step also introduces additional waiting time, because the circuit response must settle after mechanical motion or electrical switching of the tuning elements, and sufficient signal integration time is needed to obtain high-SNR reflection-coefficient feedback. As a result, iterative search methods suffer from a large number of iterations, long tuning time, and limited adaptability across frequency bands. In cases of significant mismatch, the number of iterations can sometimes reach dozens.

An NMR resonator is a near-field magnetically coupled loop antenna, and the basic principle of its tuning and matching is consistent with that of other antenna resonators. To search for optimal solutions more efficiently in complex discrete state spaces, some studies have turned to intelligent optimization strategies based on metaheuristic algorithms. For example, Bito et al. proposed an active matching technique with real-time genetic-algorithm control for antennas used in biomedical wireless power transfer systems [9]. Although evolutionary algorithms offer advantages over traditional linear scanning when dealing with nonlinear and multimodal optimization problems, their core mechanism still relies on iterative cycles of population generation, fitness evaluation, and mutation. In addition to these methods, particle swarm optimization [10], steepest descent [11], fuzzy algorithms [12], and several other approaches reported in [13,14,15] have also been explored. In recent years, neural network-based machine learning methods trained on large datasets have been introduced for automatic tuning and matching of antenna resonators to satisfy the demand for intelligent and rapid detection. To reduce the high time cost caused by repeated stepping and measurement in hardware-in-the-loop search, machine learning has been incorporated into automatic tuning and matching [16,17,18,19,20,21,22,23]. For instance, Li et al. proposed an automatic impedance-matching method based on a feedforward backpropagation (BP) neural network [16]. By offline training, the method learned a nonlinear mapping between equivalent load impedance and the optimal matching-capacitor values, thus converting traditional online optimization into efficient direct inference and improving execution efficiency by 108.5% relative to a genetic algorithm. Jeong et al. further applied neural networks to adaptive impedance matching of wireless power-transfer antennas [17] and proposed a hybrid control strategy that combines circuit-parameter prediction with hardware-topology switching. Their offline-trained feedforward neural network simultaneously outputs capacitor-control commands and optimal transmit-coil selection within milliseconds based on the measured impedance features.

However, these methods are not directly applicable to automatic tuning and matching of NMR resonators. Firstly, practical NMR resonators are multi-frequency inner–outer dual-coil structures. Electrical characteristics, such as decoupling networks between coils, isolation networks between frequency points, and parasitic effects between the coils and the samples, make the network topology highly complex and diverse. In addition, NMR experiments are affected by a variety of dynamic disturbances, including frequency drift caused by magnetic field drift, component performance changes under variable temperature testing, and device aging during long-term continuous acquisition. Meanwhile, the samples placed inside the NMR resonator coil are frequently replaced during NMR measurements, leading to frequent changes in load impedance. Some highly concentrated ionic samples induce large dynamic variations in both resonant frequency and matching condition. Recent developments in in situ NMR detection of high-concentration ionic samples have further raised the requirements for fast real-time ATM. These factors pose substantial challenges to ATM. Conventional neural networks are usually task-specific models and tend to suffer from performance degradation when transferred across structures and frequencies, making them insufficient for multi-nuclear, multi-coil, and high-throughput rapid NMR experiments. Moreover, training a high-accuracy neural network typically requires large volumes of measured data that cover all possible loading conditions. In practical NMR environments, collecting large-scale datasets from different samples for each coil type is both time-consuming and costly. As a result, previously reported neural-network-based methods are difficult to adapt rapidly when the experimental protocol, sample type, or system characteristics change and only a small number of real measured samples are available. Finally, most existing studies directly use mean squared error in parameter space as the loss function and do not account for the extremely high sensitivity near high-Q resonance points in NMR coils. Physically, even a small capacitor prediction error may lead to severe deterioration of S_11_, and a generic loss function without physical constraints is inadequate for guiding the neural network toward this nonlinear safety boundary.

Among the data-efficient modeling and optimization methods relevant to this work, Gaussian process regression [24], Bayesian optimization [25], neural process variants [26,27], and physics-informed neural networks [28] are all valuable points of reference. Gaussian process regression is a natural few-shot baseline, but standard GP modeling is more naturally suited to single-task regression with a fixed input structure, whereas the scenario considered here involves mixed-condition inputs, multiple circuit topologies, multiple resonance frequencies, and task-level adaptation based on historical tuning tasks. Bayesian optimization is highly effective for expensive black-box optimization problems, but it remains fundamentally an iterative online search strategy, while this work aims to reduce repeated trial-and-measure cycles on real hardware by learning a transferable conditional predictor. Neural process variants such as NP [26] and ANP [27] may offer stronger expressive power, but the present study places greater emphasis on model simplicity, training stability, and practical deployability. PINN-based methods are also related in spirit to this work; however, the practical NMR probe system considered here does not admit a sufficiently accurate and unified governing equation, and is therefore not well suited to full residual-based physics supervision. Under these engineering constraints, CNP provides a more suitable balance for automatic tuning and matching of NMR probes.

To address the above issues, this paper proposes a physics-informed few-shot learning method for automatic tuning and matching that targets wideband multi-resonance-frequency operation and real-time sample states, and systematically integrates CNP with the problem of automatic tuning and matching for NMR probes for the first time. The proposed method is developed for an application setting with distinctive engineering characteristics, including scarce measured data, high sample acquisition cost, multiple circuit topologies, and multiple resonance frequencies. To accommodate this scenario, the present work jointly designs the problem formulation, input–output representation, and physics-informed loss function around the task of automatic tuning and matching for NMR probes. Specifically, the proposed method uses a conditional neural process (CNP) [29] as the core predictor and formulates NMR RF probe tuning and matching as a function-regression problem conditioned on circuit structure and operating frequency. By learning a shared prior at the task-distribution level, the model can rapidly infer the optimal configuration of tunable components under different loading conditions based solely on a small number of actual real-machine context measurements, thereby reducing the burden of repeated search and measurement in traditional closed-loop tuning. On top of this, this work develops a physics-informed loss tailored to the NMR automatic tuning and matching problem. Instead of using a generic empirical weighting strategy, the proposed loss is constructed from the local sensitivity of S_11_ and a frequency-offset term, so that the model places stronger emphasis on mismatch-sensitive directions under high-Q and narrowband operating conditions. This design improves the consistency between statistical learning and the physical objective of NMR tuning and matching. Based on this framework, we perform simulation analyses of model parameters and performance, compare different methods under different numbers of sampling points and NMR samples, and build an experimental automatic tuning-and-matching system for NMR probes. Real-probe validation is then carried out over multiple circuit topologies, multiple resonance nuclei, and wideband multi-target scenarios. The results show that, compared with conventional BP neural network-based methods, the proposed approach achieves tuning and matching that are closer to the desired target and enables accurate automatic tuning and matching across different circuit topologies and multiple resonance nuclei using only a small number of NMR samples. The proposed method consistently improves both tuning accuracy and the number of real measured training samples required per task, and offers a more favorable accuracy–cost tradeoff, especially in cross-topology and cross-frequency scenarios. It therefore provides an efficient solution for emerging NMR techniques and applications such as rapid in situ real-time detection and automatic tracking of tuning and matching.

The main contributions of this work are summarized as follows:

- We formulate NMR automatic tuning and matching as a structure- and frequency-conditioned few-shot regression problem and develop the first meta-learning method specialized for the NMR ATM scenario.
- We design a physics-informed loss based on the local sensitivity of S_11_ and frequency-offset regularization, which improves learning consistency with the physical objective of high-Q NMR tuning and matching.
- We develop a unified prediction framework for limited-sample, cross-topology, and cross-frequency NMR tuning tasks through task-conditioned modeling and application-oriented input organization.
- We validate the proposed method through both simulation and hardware experiments, demonstrating its feasibility and effectiveness in practical NMR probe automatic tuning and matching.

## 2. Analysis of Automatic Tuning and Matching Principles Based on Neural Networks

### 2.1. Principles of Automatic Tuning and Matching in NMR Systems

**Figure 1:**
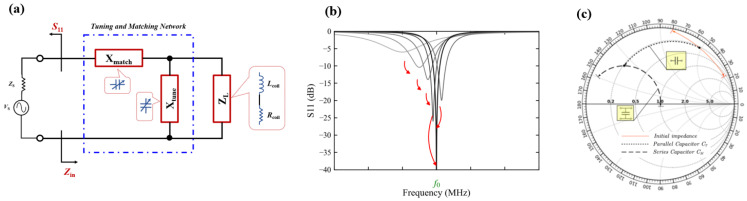
Principles and conventional methods of tuning and matching for NMR probes (**a**) Block diagram of tuning and matching; (**b**) Schematic of search-based automatic tuning and matching, where the red arrows indicate the movement of the S_11_ resonance dip during the iterative search process toward the target frequency *f*_0_; (**c**) Smith chart representation of tuning and matching.

As shown in Figure 1a, an NMR resonator is a single-port L-C network. During transmission, its input is connected to an RF power amplifier; during reception, it is connected to a low-noise amplifier. The inductance L of the NMR resonator acts as a near-field antenna. The sample is placed inside L and, after electromagnetic excitation, emits NMR signals that are received by L. Unlike other antenna resonators, the state of NMR samples varies substantially, resulting in significant changes in the filling condition of the inductance L and, consequently, continuously varying load impedance. Therefore, tuning and matching must be performed whenever the sample is changed during testing. According to the maximum power transfer theorem, the system achieves optimal energy-coupling efficiency and minimum reflection coefficient S_11_ only when the input impedance of the NMR resonator is conjugately matched to the characteristic impedance of the transmission line:

(1) $$\Gamma =\frac{Z_{coil}-Z_{0}}{Z_{coil}+Z_{0}}$$

where $Z_{coil}$ denotes the reactance of the inductance L and can be written as $Z_{coil}=R_{coil}+j\omega L_{coil}$, where $L_{coil}$ is the coil inductance and $R_{coil}$ is the equivalent series resistance.

The coil of an NMR resonator usually requires low inductance and low series resistance. Therefore, the goal is to transform $Z_{coil}$ into a pure resistance with $Z_{0}=50\;\Omega$ while ensuring that the circuit is in resonance at the target Larmor angular frequency $\omega_{0}$. This joint process is referred to as tuning and matching. An NMR resonator achieves tuning and matching by changing the impedance values of series and shunt components in the network, typically by adjusting two capacitors. The sample to be tested is placed inside the space enclosed by the inductance L and becomes the filling medium of L, which causes the impedance of L to vary with the sample. Ionic saline samples in particular can induce dramatic changes in both reactance and resistance, leading to severe shifts in resonant frequency and strong input impedance mismatch.

As shown in Figure 1b, iterative search-based automatic tuning and matching takes the matching dip of the input reflection-coefficient curve S_11_ near resonance as the search target and adjusts the capacitor values in the matching network so that the dip gradually approaches the resonance point and eventually reaches a satisfactory match. This process requires continuous iteration and consumes substantial time. When the S_11_ dip is not pronounced or falls outside the search range, the search may fail to converge and must restart from the initial values by exhaustive traversal. Some improved iterative-search methods reduce the number of search steps by establishing a lookup table between impedance states and capacitor values. However, for impedance states not covered by the table, such as those arising from new experimental protocols or changed sample states, the search still needs to start from the initial point and proceed by traversal. For multi-nuclear NMR resonators, the S_11_ dip may even fall outside the search range when switching across frequency regions.

From the perspective of network structure, the tuning and matching of NMR coils can be regarded as a nonlinear constrained optimization problem, where a set of adjustable capacitors $\left(C_{T},C_{M}\right)$ are sought to minimize the expression (1) under varying load impedances $\left(R_{L},X_{L}\right)$, given a fixed circuit structure **s** and a fixed resonant frequency *ω*. That is, $f:\left(R_{L},X_{L},\omega ,s\right)\to \left(C_{T},C_{M}\right)$. This process requires the measured input impedance *Z_in_* to determine the circuit–network relationship between the input and the loaded coil, including couplings that cannot be accurately represented by an equivalent circuit. Based on this relationship, the element values of the matching network that yield a 50 Ω input impedance for different sample loads can then be determined. Figure 1c illustrates the impedance trajectory of a typical L-type matching network on the Smith chart, where the initial load point is mapped toward the chart center. Once the network relationship is known, the two capacitors can in principle be directly adjusted along the impedance-matching path to reach the resonance and matching point. In practice, however, actual NMR resonator coils are inner–outer dual-layer structures with highly complex mutual coupling and parasitic effects. The analytical circuit equations therefore become high-order nonlinear equations that are difficult to solve. Some ionic samples also introduce additional reactances, which require unknown correction terms in the circuit equations. These factors make it difficult to obtain an accurate equivalent circuit and precise network parameters for the true topology.

To address these challenges, we introduce neural networks into automatic tuning and matching of NMR resonators. By learning from sample data, the neural network adaptively captures the circuit network and establishes the relationship among input impedance, the loaded coil, and the tuning/matching elements. Because no standard training set exists and it is impossible to collect large-scale data from many real NMR samples within a short time, especially for experiments with a large dynamic range, automatic tuning-and-matching methods developed for other antennas and trained on massive datasets cannot satisfy the requirements of adaptive NMR autotuning. We therefore propose an improved few-shot neural-network method that can continue learning during training, gradually adapt to different samples, and improve prediction accuracy.

### 2.2. Function-Regression Modeling and Neural Network Design for NMR Automatic Tuning and Matching

**Figure 2:**
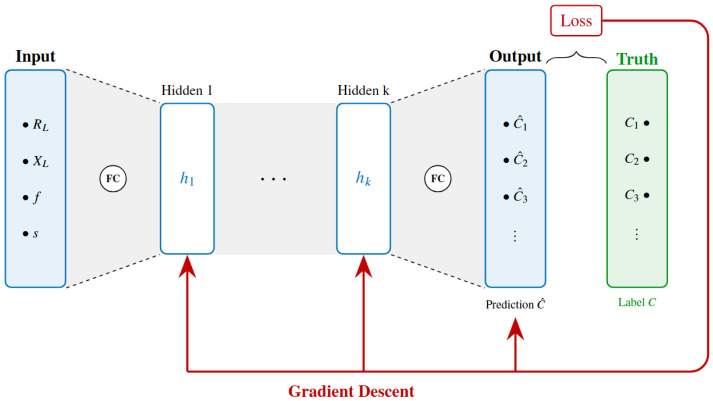
Schematic illustration of the neural network-based automatic tuning and matching task as a supervised regression problem.

In traditional analytical methods or search algorithms, the mapping from the measured state $\left(R_{L},X_{L},\omega ,s\right)$ to the optimal capacitor settings $\left(C_{T},C_{M}\right)$ is usually obtained either through explicit formula derivation or through iterative search in a discrete capacitor-combination space. Here, we view tuning and matching in a practical NMR probe as consisting of two mappings. The first is the mapping from input impedance to load impedance, which reflects the structure and electrical parameters of both the tuning-and-matching circuit and the loaded resonant circuit. The second is the mapping from the load impedance corresponding to 50 Ω input impedance to the tuning-and-matching capacitor values. In other words, after obtaining the load impedance through the transmission characteristics of the circuit system from the first mapping, one solves for the circuit parameters $\left(C_{T},C_{M}\right)$ that yield 50 Ω input impedance. In practical circuits, the ideal network relationship is significantly altered by factors such as decoupling networks, isolation and filtering networks, feed lines, parasitic parameters, and the filling medium inside the coil. As a result, analytical formulas are difficult to use for accurately characterizing the real system. On the other hand, the load impedance is determined by the properties of different NMR samples and is difficult to know precisely. We therefore merge the above two mappings into a direct mapping from input impedance to the capacitor settings that yield a 50 Ω match. Under this formulation, tuning and matching can be more naturally viewed as a data-driven function-regression problem. As discussed in the previous subsection, the tuning-and-matching process is essentially a nonlinear mapping problem: for any given load $Z_{L}$ impedance and operating frequency ω, there exists an optimal capacitor combination $C_{opt}=\left(C_{match,opt},C_{tune,opt}\right)$ that matches the input impedance to 50 Ω. Specifically, the input feature vector for each tuning task can be defined as x= $\left[R_{in},X_{in},{C_{1}}^{\prime },\ldots ,{C_{N_{max}}}^{\prime },f,s\right]$ using the measured real parts $R_{in}$ and imaginary parts $X_{in}$ of the equivalent input impedance under the current loaded sample, adjustable component group $\left[{C_{1}}^{\prime },\ldots ,{C_{N_{max}}}^{\prime }\right]$ before tuning and matching, the target resonance frequency f (which may correspond to multiple Larmor frequencies such as ^1^H and ^13^C), and one-hot vector **s** encoding the circuit structure category, coil channel, or other hardware configuration. The output is a vector of tunable-element states $C=\left[C_{1},\ldots ,C_{N_{max}}\right]$ with a unified maximum dimensionality $N_{max}$, and nonexistent elements are ignored by a structural mask. The selected input features are chosen to reflect the key task conditions of NMR automatic tuning and matching. The measured input impedance provides the most direct electrical description of the current load state and circuit response under the present sample condition. The target frequency is included because different resonance nuclei and operating channels correspond to different matching objectives and sensitivity regimes. The structural encoding is introduced to distinguish different matching-network topologies within a unified model. In this way, the model can learn shared representations across related tasks while still preserving topology-specific conditioning information. By using traditional manual tuning or automatic search algorithms, one can collect offline sample pairs $\{\left(x^{\left(i\right)},C_{*}^{\left(i\right)}\right){\}}_{i=1}^{M}$ to form a training set, where the measured input impedance X(i, j) is recorded while the capacitor values C(j) are traversed over their allowable ranges. The approximately optimal capacitor combination $C_{*}^{\left(i\right)}$ is obtained after sufficient tuning under each structural condition. The neural network is then trained to approximate this unknown mapping:

(2) $${\hat{C}}^{\left(i\right)}=f_{\theta }\left(x^{\left(i\right)},\;C(j)\right)$$

where $f_{\theta }\left(\cdot \right)$ is composed of several fully connected layers and nonlinear activation functions, such as ReLU or tanh, and the most common loss function is the mean squared error:

(3) $$L\left(\theta \right)=\frac{1}{M}\sum_{i=1}^{M}\sum_{k=1}^{N_{max}}m_{k}^{\left(i\right)}{\left({\hat{C}}_{k}^{\left(i\right)}-C_{*,k}^{\left(i\right)}\right)}^{2}$$

where $m_{k}^{\left(i\right)}\in \{0,1\}$ is the structural mask used to suppress components that do not exist in the i-th structure (for example, an L-type network has two tunable elements, whereas a Π-type network has three). This design allows the model to share parameters across different circuit structures without forcing nonexistent components to contribute to the training objective. As a result, the mask improves the consistency of cross-topology learning and reduces interference from invalid output dimensions. Figure 2 illustrates the BP neural network-based modeling scheme constructed for NMR automatic tuning and matching: the input layer receives the real and imaginary parts of the input impedance, the adjustable component group, the frequency, and the structural encoding. In multi-task scenarios, environmental parameters such as temperature and port index can also be included. The middle layers consist of several fully connected hidden layers with nonlinear activations, which learn complex nonlinear mappings and shared features across structures. The output layer is a masked multidimensional regression head corresponding to the tunable capacitor values that achieve 50 Ω input impedance for different sample loads. During inference, once measurements under the current loading condition are obtained, the neural network can directly provide an initial capacitor setting $\hat{C}$ as the starting point for a small local search or even single-step application, thereby greatly reducing the number of point-by-point search iterations.

## 3. Automatic Tuning and Matching Method Based on Physics-Informed Conditional Neural Processes

### 3.1. Automatic Tuning and Matching Method Based on CNP

**Figure 3:**
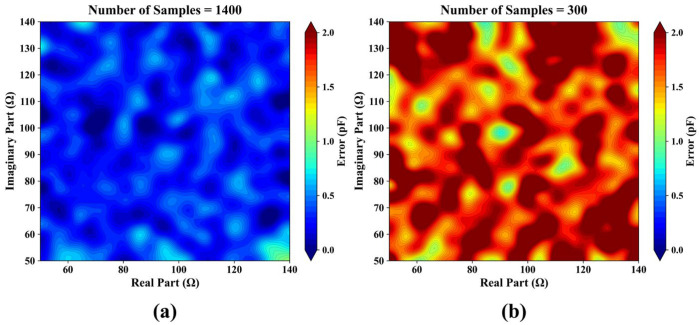
Prediction error distributions of a conventional BP neural network for an L-type matching circuit under different numbers of samples: (**a**) number of samples is 1400 and (**b**) number of samples is 300.

Although previous neural network-based studies have removed the time cost of iterative search, they still exhibit significant limitations. A neural network implicitly encodes the physical structural information of the circuit into its weight parameters θ and essentially fits a static manifold associated with a fixed circuit topology. This means that once the hardware environment of the NMR system changes, for example due to parameter drift caused by component aging, the prediction may fail because the underlying functional mapping has changed while the fixed network weights cannot perceive such variation. As shown in Figure 3, a single model usually requires a large amount of training data to learn a satisfactory initialization, which is difficult to obtain in practical NMR applications because data acquisition is expensive. To overcome the limitations of treating automatic tuning and matching as a static function-regression problem, this paper proposes an NMR automatic tuning and matching method based on conditional neural processes (CNP).

CNP is a few-shot function-regression model that combines neural networks with stochastic processes. Its core idea is to learn a conditional function prior over a distribution of tasks. Given a small number of contextual observations $D_{C}=\{\left(x_{i},y_{i}\right){\}}_{i=1}^{K}$, a CNP predicts the output distribution $p\left(y^{*}|x^{*},D_{C}\right)$ of arbitrary target inputs $x^{*}$ in the same task through a shared encode–aggregate–decode architecture. In this work, we formulate NMR automatic tuning and matching as a conditional probabilistic inference problem. We assume that the current physical state of the NMR resonator corresponds to an unknown optimal tuning-and-matching function, and the goal is to predict the optimal capacitor values for target inputs given only a small number of observations $D_{C}=\{\left(x_{i,C},C_{i,opt}\right){\}}_{i=1}^{K}$ (Support Set). For a tuning task $T_{s,f}$ defined under a particular circuit structure **s** and resonance frequency *f*, the task contains a series of samples $\left\{\left(x_{n},C_{n,opt}\right)\right\}$ under different load states, where we have the following input feature vector:

(4) $$x_{n}={\left[R_{in},X_{in},{C_{1}}^{\prime },\ldots ,{C_{N_{max}}}^{\prime },f,s\right]}^{⊤}$$

consisting of the input impedance $Z_{in}=R_{in}+jX_{in}$, the adjustable component group $\left[{C_{1}}^{\prime },\ldots ,{C_{N_{max}}}^{\prime }\right]$, the target frequency *f*, and the structural one-hot code **s**; the output $C_{n,opt}\in R^{N_{max}}$ is a capacitor vector of unified length, and a structure-dependent mask $m_{s}$ indicates which components are valid under the current structure **s**. In each CNP training iteration, one task is randomly sampled from the task set $\left\{T_{s,f}\right\}$, and the samples in that task are randomly split into context set and target set:

(5) $$D_{C}=\{\left(x_{i,C},C_{i,opt}\right){\}}_{i=1}^{K},D_{T}=\{\left(x_{j,T},C_{j,opt}\right){\}}_{j=1}^{M}$$

The model consists of three parts: encoder, aggregator, and decoder. The encoder $h_{\phi }\left(\cdot \right)$ of the CNP first maps each context sample into a representation vector:

(6) $$r_{i}=h_{\phi }\left(x_{i,C},C_{i,opt}\right)$$

A permutation-invariant aggregation operator, implemented here as simple averaging, is then used to obtain a task-level representation:

(7) $$r=aggregator\{r_{i}{\}}_{i=1}^{K}=\frac{1}{K}\sum_{i=1}^{K}r_{i}$$

This vector can be interpreted as a function prior summarized from a small number of context points under the given structure **s** and frequency conditions *f*. The decoder $g_{\theta }\left(\cdot \right)$ then jointly maps each target input $x_{j}^{T}$ and the task representation r to produce the predicted tunable-capacitor values:

(8) $${\hat{C}}_{j}=g_{\theta }\left(x_{j,T},r\right)$$

**Figure 4:**
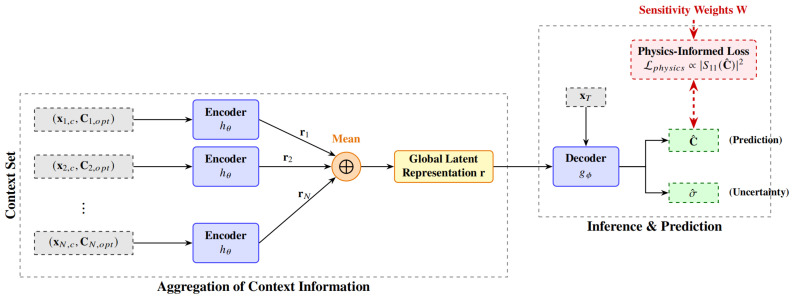
Schematic of the physics-informed conditional neural process framework for automatic tuning and matching.

In implementation, both the encoder and the decoder adopt multi-layer perceptron structures, and the structural one-hot code and frequency information are explicitly concatenated at the input. This choice is motivated by the need for a lightweight and stable function approximator that can flexibly handle mixed continuous and discrete task descriptors. The aggregation module uses simple mean pooling to preserve permutation invariance of the context set while keeping the task representation compact and robust for few-shot adaptation. As illustrated in Figure 4, the complete automatic tuning-and-matching process under the CNP framework can be described as follows. During offline training, a large number of historical tasks $\left\{T_{s,f}\right\}$ are sampled episodically. For each task, a small subset of samples is randomly selected as the context set $D_{C}=\{\left(x_{i,C},C_{i,opt}\right){\}}_{i=1}^{K}$ and the remaining samples are used as the target set $D_{T}$. The model outputs predictions for the target points and minimizes the negative log-likelihood or mean squared error over all tasks and target samples, thereby learning a conditional family of functions $f_{\theta ,\phi }:x\mapsto C$ that generalizes across structures and frequencies. During online deployment, for a new loading condition, one first measures the input impedance under the current structure and frequency. Historical data under the same structural and frequency conditions are then used to obtain a task-specific representation through encoding and aggregation. Finally, a single forward inference is performed on the input feature corresponding to the current tuning-and-matching state, i.e., the measured input impedance together with the structure and frequency identifiers. A small amount of local search may then be added if necessary for final fine-tuning.

### 3.2. Design of a Physics-Informed Hybrid Loss Function Based on Reflection-Coefficient Sensitivity

**Figure 5:**
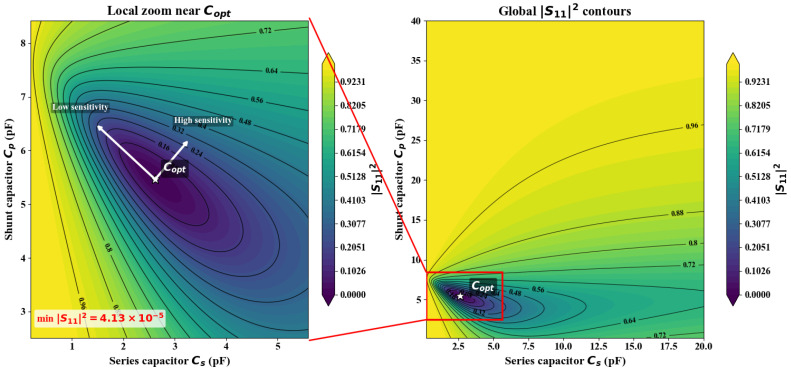
Local and global contour maps of |S_11_|^2^ in the capacitor space for an L-type matching circuit under fixed load impedance.

Although the CNP architecture provides strong meta-learning capability, training it with only standard mean squared error loss $L_{MSE}=∥C_{pred}-C_{gt}{∥}_{2}^{2}$ is insufficient, because regression error in capacitor values alone cannot fully reflect the true matching quality or engineering risk in practical NMR resonators. As illustrated in Figure 5, in particular, near the high-Q narrowband operating point required by NMR resonators, the effect of capacitor perturbations on the reflection coefficient S_11_ is highly nonlinear, so the model may learn a statistically average optimum that is not physically optimal. In high-Q narrowband resonant circuits, even a very small capacitor error may cause a sharp degradation in S_11_. To incorporate this physical prior into network training, we design a physics-informed hybrid loss function.

According to microwave network theory, the goal of tuning and matching is to minimize the reflected energy at the operating frequency. For a given task $T_{s,f}$, let $C_{opt}$ denote the optimal capacitor vector obtained through high-precision search under a specific load condition. The reflected power at the operating frequency $\omega_{0}$ can then be written as:

(9) $$F\left(C\right)={|\begin{array}{c} S_{11}\left(C\right) \end{array}|}^{2}$$

Because the explicit expression of ${|\begin{array}{c} S_{11}\left(C\right) \end{array}|}^{2}$ differs across circuit structures, we perform a second-order Taylor expansion so that the loss becomes generic to arbitrary circuits while remaining differentiable with respect to the capacitor values. Considering that ${|\begin{array}{c} S_{11}\left(C\right) \end{array}|}^{2}$ reaches a minimum value (close to 0) at the optimal matching point $C_{opt}$, its local behavior is mainly determined by the second-order term:

(10) $${\left|S_{11}\left(C\right)\right|}^{2}\approx \frac{1}{2}{\left(C-C_{opt}\right)}^{T}H\left(C-C_{opt}\right)$$

where **H** is the Hessian matrix with respect to the capacitor vector, and its elements $H_{ij}={\left.\frac{\partial^{2}{\left|S_{11}\right|}^{2}}{\partial C_{i}\partial C_{j}}\right|}_{C=C_{opt}}$ characterize the sensitivity, i.e., the curvature, of the reflection coefficient to capacitor perturbations. In high-Q tasks, the S_11_ curve is sharper and the eigenvalues of **H** are larger. In low-Q broadband tasks, the curve is flatter and the corresponding eigenvalues are smaller. In engineering implementation, the number of capacitors varies across structures, and directly computing the full Hessian matrix is computationally expensive. In practical NMR resonator matching tasks, our main objective is to capture the magnitude of sensitivity under different physical scenarios rather than the exact geometric curvature directions. The off-diagonal terms of the Hessian represent coupling effects between simultaneous adjustments of two elements. By contrast, near resonance, the mismatch caused by perturbing a single element alone is usually more pronounced than the coupling induced by simultaneous small changes in two elements. Therefore, the diagonal terms dominate the energy of the Hessian matrix. To further assess the validity of the diagonal approximation, we quantify the relative contribution of the off-diagonal Hessian terms using the following ratio:

(11) $$\rho =\frac{∥H_{off}{∥}_{F}}{∥H_{diag}{∥}_{F}}$$

where $H_{diag}$ and $H_{off}$ denote the diagonal and off-diagonal parts of the local Hessian, respectively, and $∥\cdot {∥}_{F}$ denote the Frobenius norm. A small value of ρ indicates that the local mismatch sensitivity is dominated by single-element perturbations, in which case the diagonal approximation captures the main sensitivity magnitude relevant to the proposed physics-informed loss. By contrast, larger values of ρ indicate stronger local coupling among tunable elements, for which the diagonal approximation becomes less accurate.

Table 1 presents the local Hessians of ${\left|S_{11}\right|}^{2}$ with respect to the tunable capacitor vector at several representative operating points, and further quantifies the relative contribution of the off-diagonal terms by the ratio ρ. For the computed L-, Π-, and T-type networks, the resulting ρ values under representative loads range from 0.0685 to 0.2081. In particular, the L-type network exhibits strongly diagonal-dominant behavior under the 20 + j40 Ω load, whereas the Π- and T-type networks show moderate, yet still subdominant, off-diagonal coupling contributions relative to the diagonal terms. These results indicate that, in most computed cases, the diagonal approximation can adequately capture the dominant local sensitivity magnitude required by the physics-informed loss proposed in this work. At the same time, we note that the T-type network yields a relatively larger ratio of ρ = 0.2081 under the 30 + j60 Ω load, suggesting that when multiple tunable capacitors jointly control the same resonance or matching mode within a local region, the off-diagonal terms of the Hessian may increase noticeably. In such cases, ρ becomes larger and the accuracy of the diagonal approximation decreases.

In this work, the diagonal approximation is adopted primarily as an engineering simplification, so that a unified and computationally efficient loss function can be constructed across different matching-network topologies. Its main purpose is to encode the dominant magnitude of local sensitivity rather than to fully recover the complete local error geometry. Based on the above analysis, and to balance engineering simplicity with cross-topology consistency, we thus simplify the Hessian into a diagonal weighting matrix $W\approx diag\left(H\right)$, where each element $W_{i}$ represents the independent contribution of the corresponding tunable capacitor to the deterioration of the reflection coefficient. This assumes that, near the operating point, the local mismatch sensitivity is mainly governed by perturbations of individual tunable elements, while the second-order coupling among tunable capacitors remains relatively weak. To obtain these values, finite-difference computation is performed and stored offline during dataset generation. Compared with Q-value estimation formulas based on ideal circuit theory, the finite-difference method can directly reflect the real circuit response, including complex parasitic effects. Accordingly, the physics-informed loss is given by:

(12) $$L_{Physics}=\frac{1}{N}\sum_{i=1}^{N}W_{i}\cdot {\left(C_{pred,i}-C_{opt,i}\right)}^{2}$$

This loss imposes a larger penalty on prediction errors that occur along directions of high curvature. This means that for a high-Q circuit, due to the presence of a sharp valley in its ${\left|S_{11}\right|}^{2}$ near $C_{opt}$, the eigenvalues of the Hessian are large, and the model is therefore forced to fit the data with much higher precision, since even a slight deviation leads to a pronounced increase in $L_{Physics}$. In contrast, for a low-Q circuit, the penalty is relatively mild. This weighting mechanism tightly couples model learning to the physical properties of the circuit and guides the network toward predictions that are more meaningful from an engineering perspective.

Another goal of tuning and matching is to make the best reflection point of the S_11_ curve within the tunable capacitor range coincide with the desired operating frequency. By the physical definition of resonance, the imaginary part of the input impedance is strictly zero at the target frequency $f_{0}$. Moreover, the frequency offset $\Delta f=f-f_{0}$ and the residual reactance $X\left(f_{0}\right)$ at the target frequency are linked by a highly linear analytical relationship: $\Delta f\approx -X\left(f_{0}\right)/\left(\frac{\partial X}{\partial f}{|}_{f_{0}}\right)$. In other words, the deviation between the actual resonant frequency and the target frequency is mathematically equivalent to the absolute value of the input-reactance residual at the target frequency. We therefore define the frequency-offset loss as the squared L2 norm of the reactance at the target frequency as follows:

(13) Lfreq=ImZinf0,Cpred/Z02

The final overall loss function is defined as:

(14) $$L_{total}=L_{MSE}+\lambda L_{Physics}+\beta L_{freq}$$

where $L_{\mathrm{MSE}}$ is the native CNP mean squared error loss, λ is the weighting coefficient of the physics loss, and β is the weighting coefficient of the frequency-offset loss.

### 3.3. System Architecture of the Proposed Method

**Figure 6:**
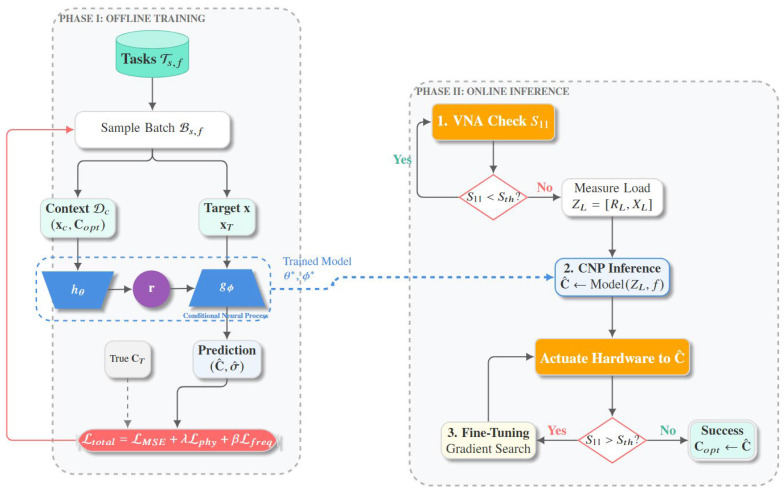
Overall system architecture of the proposed physics-informed CNP-based automatic tuning and matching method.

Based on the aforementioned theory, the system architecture diagram of our proposed method is illustrated in Figure 6.

## 4. Results

**Figure 7:**
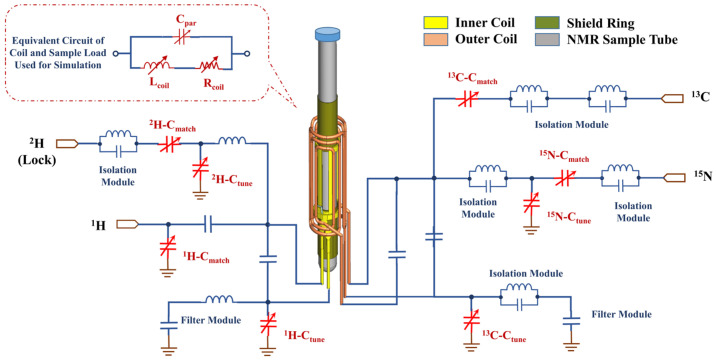
Equivalent circuit of the NMR probe.

To demonstrate the effectiveness of the proposed method, we conduct both simulations and real NMR probe experiments. In the simulations, the probe coil and the tuning-and-matching circuit are replaced with an equivalent circuit model of the actual system, as shown in Figure 7. The load is represented by an equivalent L-R-C model, where $L_{coil}$ and $R_{coil}$ denote the equivalent inductance and resistance of the coil, respectively, and $C_{par}$ is the equivalent parasitic capacitance in parallel with the coil. Other coupling and parasitic effects that cannot be accurately represented in the practical system are ignored in simulation. These omitted effects are automatically incorporated during learning in the real experimental system. The simulations are intended mainly to validate the sample requirements of the proposed method and the effectiveness of the physics-informed loss function.

### 4.1. Dataset Construction

Following the proposed method, we construct the dataset by traversing the tuning-and-matching capacitor values under different load conditions. The dataset inputs include the load impedance (sample), the capacitor values within the tunable range of the tuning capacitor, the capacitor values within the tunable range of the matching capacitor, and the measured input impedance. The dataset output is the set of tuning-and-matching capacitor values that transforms the input impedance to 50 Ω under each load impedance. To make the simulation experiments better approximate the complex operating conditions of real NMR probes, we do not use a single-distribution data-generation strategy. Instead, we establish differentiated parameter sampling spaces according to the physical characteristics of different nuclei under practical circuit topologies. Figure 7 shows the equivalent circuit representation of a practical tri-resonance NMR probe topology, including four independent channels: ^1^H, ^13^C, ^15^N, and ^2^H. Based on the structural characteristics of practical NMR coils, the no-load equivalent impedance parameters and tunable capacitance ranges for different channels are listed in Table 2.

In the simulations, Gaussian random perturbations of ±50% and ±50% are applied to the coil inductance $L_{coil}$ and parasitic capacitance $C_{par}$ around their nominal values under different load impedances so as to cover variations in the dielectric properties of the samples. Dataset generation strictly follows the topological constraints of the practical circuits. For each randomly generated load impedance $Z_{load}$, the optimal capacitor combination $C_{opt}=\left(C_{tune},C_{match}\right)$ for each channel in Figure 7 is solved using the ADS circuit simulator. If a given load condition requires capacitor values outside the physical tuning range, that sample is labeled as unmatched and removed to ensure the validity of the training data. In the simulations, NMR samples are represented in the form of complex impedance.

Although the simulation data are generated from an equivalent-circuit model rather than from a full electromagnetic or in-magnet hardware model, several steps were taken to improve their engineering relevance. Specifically, the circuit parameters were chosen according to practical probe-channel settings, different resonance channels were modeled separately, tunable-capacitance ranges were constrained by physically realizable values, and random perturbations were introduced to cover variations in loading conditions. In this sense, the simulated data are intended to reflect the main tuning-and-matching behavior of practical NMR probe circuits. At the same time, we acknowledge that the simulated data do not fully reproduce all nonideal factors in a real NMR system, such as inter-channel coupling, unmodeled parasitics, magnetic-field-related effects, measurement noise, and hardware actuation errors. Therefore, the role of simulation in this work is to provide a controllable environment for method analysis and comparison, while the hardware experiments are used to assess practical feasibility under real operating conditions.

### 4.2. Hyperparameter Optimization and Evaluation Metrics

Model performance is not evaluated solely by numerical convergence of the loss function; instead, the ultimate tuning-and-matching effectiveness is taken as the core criterion. Specifically, a test case is regarded as successful when both the magnitude of the reflection coefficient $\left|S_{11}\right|<-30\;\mathrm{dB}$ and the distance between the best reflection point and the operating frequency point satisfy the matching requirement ($∥f_{S11}-f_{0}∥\;<1\;\mathrm{kHz}$) after tuning and matching. The matching success rate is then defined as the proportion of successful test cases among all test cases, and the final hyperparameter combination is selected to maximize this success rate. Hyperparameters are chosen using grid search. Table 3 lists the hyperparameter settings used in the simulation study, including the shared settings, the proposed method, and the BPNN baseline used for comparison. Because the proposed method involves many hyperparameters, we do not further report separate experiments for each of them individually. To ensure a fair comparison among methods, a separate BPNN model is independently optimized for each channel, whereas the proposed method adopts a unified model across channels. The final configuration is determined through a combination of preliminary experiments and grid search, with attention to matching success rate, training stability, and consistency across channels, and the resulting settings or search ranges used in subsequent experiments are directly reported. After the final configuration was selected, the same setting was kept fixed in the subsequent experiments under different sample sizes and different channels in order to fairly evaluate performance under varying data regimes, so that the comparison would reflect the intrinsic behavior of the method rather than task-specific hyperparameter retuning. We also note that the performance is naturally influenced by architectural and optimization hyperparameters; however, within the tested range, the main performance trends reported in this work remain stable, and the overall conclusions are not altered by moderate changes in these settings.

To eliminate performance fluctuations caused by differences in the computing environment and to ensure rigor and reproducibility, all model training, simulations, and comparative experiments reported in this paper are conducted on a unified computing platform. The hardware uses a single NVIDIA GeForce RTX 4060 GPU (NVIDIA Corporation, Santa Clara, CA, USA) as the acceleration device. The deep learning framework is PyTorch 1.13.1, while the numerical computation and signal processing modules rely on NumPy 1.23 and SciPy 1.9. Simulation-data generation and preprocessing are automatically completed through the interaction interface between ADS 2024 and Python 3.9.

### 4.3. Algorithm Simulations

#### 4.3.1. Simulation Experiments on Different Sample Sizes

The training samples used by the proposed method are formed from different NMR samples, the corresponding changes in tuning-and-matching capacitor values, and the measured input impedance. Therefore, the final network accuracy is affected by both the number of NMR samples and the number of capacitor sampling points. In a practical NMR system, acquisition of the capacitor values and the corresponding input impedance is very easy and incurs very little time cost. Changes in capacitor values correspond directly to motor step counts, while the input impedance can be obtained from the quadrature-detection voltage of the tuning module in the NMR system. Consequently, such data can be collected rapidly and in large quantities. In contrast, preparation of NMR samples is much more expensive and time-consuming. Therefore, from the perspective of training-data volume, reducing the required number of NMR samples is the main consideration.

**Figure 8:**
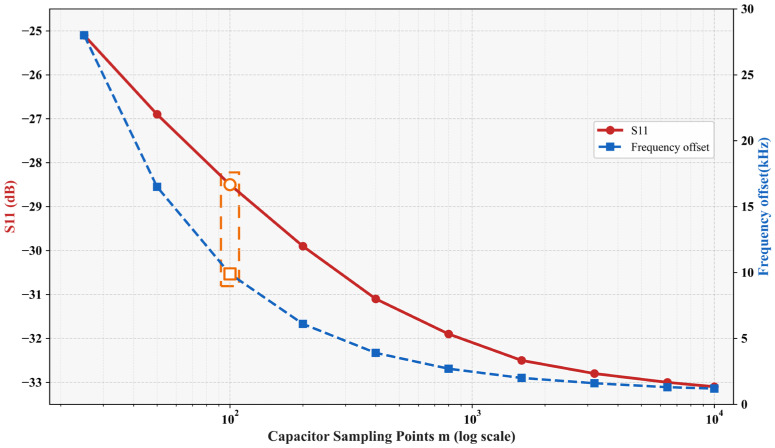
Effect of the number of capacitor sampling points on the ^1^H channel. The orange dashed box marks the representative result obtained with 100 sampling points.

To verify the effectiveness of the proposed method under few-shot conditions, we first conducted simulation experiments using different numbers of tuning-and-matching capacitor sampling points for the same load samples. The target frequency is 600 MHz. The total number of sampled points for the tuning capacitor $C_{tune}$ and matching capacitor $C_{match}$ was set to 25, 50, 100, 200, 400, 800, 1600, 3200, 6400, and 10,000. Different initial capacitance values of $C_{tune}$ and $C_{match}$, combined with various load samples, result in distinct input impedances. After training, we test the minimum reflection value of $S_{11}$ in the ^1^H channel and its offset from the target frequency. As shown in Figure 8, as the number of sampling points increased from 25 to 10,000, both S_11_ and the frequency offset improved monotonically and quickly approached saturation. The measured S_11_ improved from approximately −25.0 dB to −33.5 dB, while the frequency offset decreased from roughly 28.7 kHz to about 1.2 kHz. After the number of capacitor sampling points reached around 10^3^, the improvement became significantly slower. The results indicate that, with only 100 sampling points, S_11_ already reaches about −28.5 dB and the frequency offset is approximately 10.2 kHz, which satisfies the tuning-and-matching requirement.

**Figure 9:**
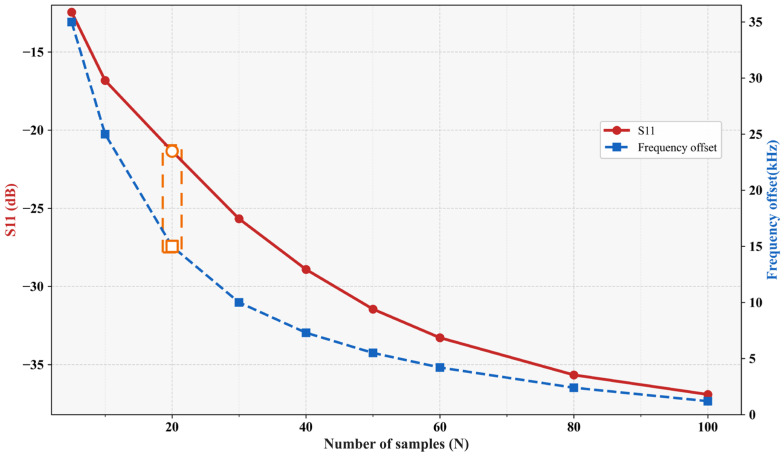
Effect of the number of training samples on the ^1^H channel. The orange dashed box marks the representative result obtained with 20 training samples.

Next, we performed simulations with the same number of tuning-and-matching capacitor sampling points while varying the number of NMR samples. Since the load impedance used to build the dataset set in simulation can be assigned arbitrarily, the number of load impedance states used in training was increased from 5 to 100 in increments of 5. The total number of capacitor sampling points was fixed at 1600. After training, we tested the minimum reflection value of $S_{11}$ in the ^1^H channel and its offset from the target frequency. As shown in Figure 9, when the number of load impedance states increased from 5 to 100, the reflection value of $S_{11}$ continuously decreased and the frequency offset was continuously reduced, with the performance gain gradually slowing in the large sample size regime. For the proposed method, S_11_ improved from −12.5 dB to −36.9 dB, while the frequency offset decreased from roughly 35.2 kHz to about 1.3 kHz. The results further show that with 20 load impedance states, the frequency offset is already around 15.3 kHz and S_11_ reaches about −21.5 dB, which is sufficient to satisfy the tuning-and-matching requirement.

#### 4.3.2. Simulation Experiments on the Impact of Key Algorithm Parameters on Performance

**Figure 10:**
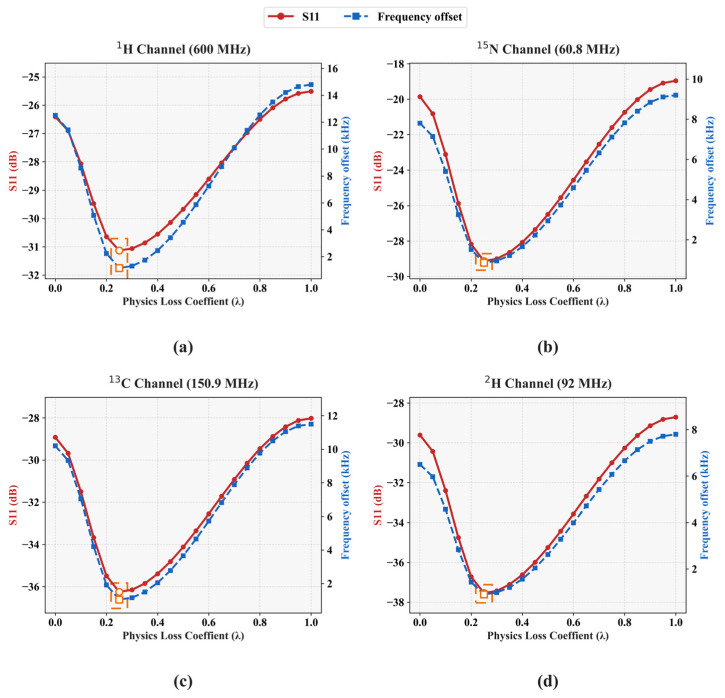
Effect of the physics-loss coefficient on S_11_ and frequency offset across four channels: (**a**) ^1^H channel at 600 MHz; (**b**) ^15^N channel at 60.8 MHz; (**c**) ^13^C channel at 150.9 MHz; (**d**) ^2^H channel at 92 MHz. The orange dashed boxes highlight the selected optimal operating point at λ = 0.25, where both S_11_ and the frequency offset achieve favorable values.

To further investigate the contribution of the physics-informed loss during model training, we compared the physics-informed loss with the standard mean squared error loss. According to (6), the balancing coefficient λ of the physics-informed loss in the proposed method was varied from 0 to 1 in steps of 0.05. When λ = 0, the physics-informed loss is disabled. During training, both the tuning-capacitor and matching-capacitor sampling points were fixed at 100, and the number of load impedance states was set to 20. The test results under different λ values are shown in Figure 10. The results indicate that model performance improves markedly and rapidly as λ increases from 0 to 0.25. When λ = 0, i.e., when the model degenerates to a native CNP without physical constraints, the lack of explicit penalization for resonance physics leads to an average matching depth of only −26.2 dB over the four channels. As the weight of the physics-informed loss is gradually introduced, the system becomes increasingly sensitive to the resonance frequency drift caused by small capacitor perturbations. In particular, when λ reaches the optimal value of 0.25, the average frequency error is compressed to about 1.1 kHz, while the average S_11_ dip depth improves significantly to −33.5 dB. This is because the native CNP, although capable of learning the statistical mapping between capacitor states and load variation, does not necessarily align capacitor error minimization with the final engineering objective of minimizing reflection in the presence of multiple equivalent topologies, parasitic parameters, and device errors in practical matching networks. The physics-informed loss acts as a strong regularizer that corrects the smoothing illusion of purely data-driven models in sparsely sampled regions and forces the neural network predictions to follow the physical laws of circuit resonance more strictly, thereby enabling accurate prediction in narrowband regions. It is worth noting, however, that the matching performance does not continue to improve monotonically as λ increases. Instead, the best performance appears around λ ≈ 0.25. When the weight becomes too large, the physical loss introduces many local minima and steep gradient valleys into the loss landscape, leading to gradient conflict during optimization. When λ is further increased to 1, the average matching depth rebounds to about −24.7 dB. Therefore, λ = 0.25 is confirmed to be the optimal operating point for this system.

**Figure 11:**
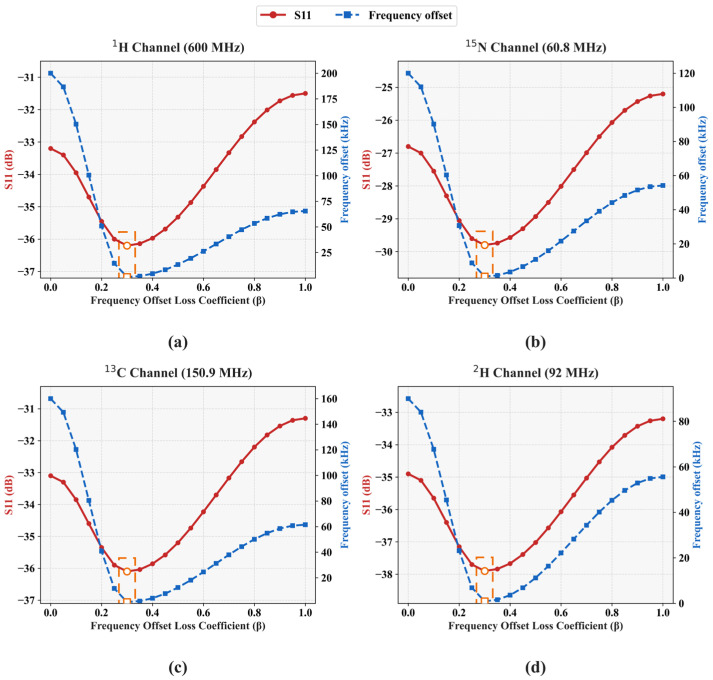
Effect of the frequency-offset coefficient on S_11_ and frequency offset across four channels: (**a**) ^1^H channel at 600 MHz; (**b**) ^15^N channel at 60.8 MHz; (**c**) ^13^C channel at 150.9 MHz; (**d**) ^2^H channel at 92 MHz. The orange dashed boxes highlight the selected optimal operating point at β = 0.30, where both S_11_ and the frequency offset achieve favorable values.

To further examine the decisive role of the frequency offset loss in center-frequency alignment, we conducted experiments on the frequency loss coefficient β while fixing the optimal physics-loss coefficient at λ = 0.25. The test results under different λ values are shown in Figure 11. When β = 0, the frequency offset loss is disabled and the system degenerates into a model driven only by amplitude optimization. The results expose the limitations of relying solely on S_11_ magnitude optimization: because pure magnitude matching cannot overcome the phase ambiguity in complex impedance space, the optimizer exhibits severe topological sliding while attempting to reduce the real-part error. As a result, although the S_11_ magnitude can still converge, the frequency deviations on the ^1^H and ^13^C channels rise sharply to about 201.1 kHz and 162.9 kHz, respectively. As the weight of the frequency offset loss is gradually increased from β = 0 to β = 0.3, the model exhibits a significant improvement in frequency alignment. When β reaches the optimal value of 0.30, the frequency offset on the ^1^H channel is compressed to about 1.2 kHz, while the S_11_ dip depth remains excellent at approximately −36.2 dB. This result indicates that the frequency offset loss forces the neural network prediction not only to approach the 50 Ω real-axis matching point, but also to pass through the zero-phase real axis in the complex plane, thereby locking the center frequency in a high-Q narrowband region. Similar to the evolution of λ, however, when β is excessively increased to 1.0, the overly large coefficient forces the model to fit small phase fluctuations too aggressively and sacrifices generalization. In that case, both the S_11_ depth and the frequency error degrade, with the latter rebounding to 65.50 kHz. Taken together, the two groups of experiments indicate that λ = 0.25 and β = 0.30 establish the best balance between physical priors and data-driven learning.

#### 4.3.3. Multi-Channel Simulation Experiments

**Figure 12:**
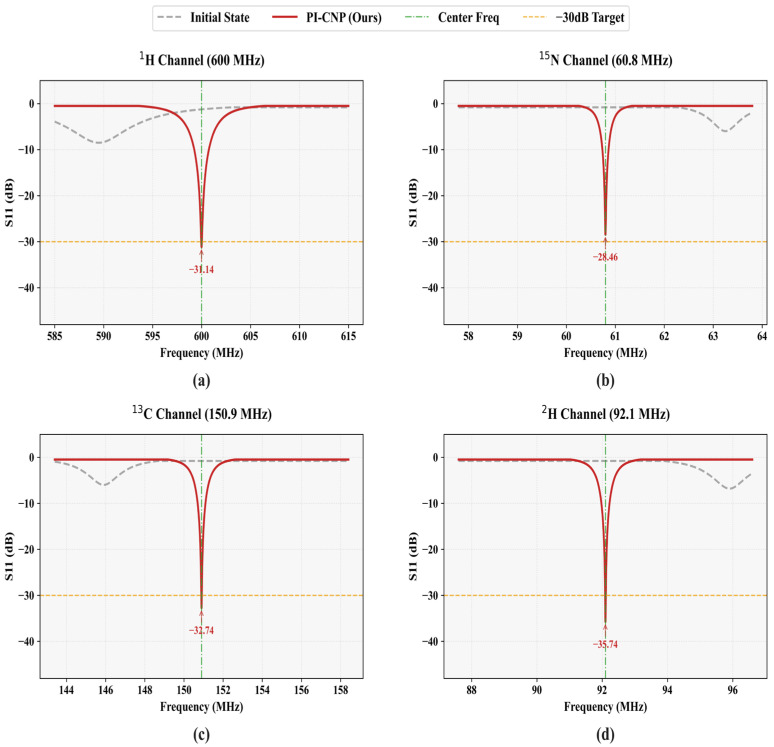
Effectiveness validation of the proposed PI-CNP method across four channels: (**a**) ^1^H channel at 600 MHz; (**b**) ^15^N channel at 60.8 MHz; (**c**) ^13^C channel at 150.9 MHz; (**d**) ^2^H channel at 92 MHz.

NMR probe heteronuclear channel targets wideband and multi-frequency operation. Because no standard training set exists for tuning and matching of NMR resonators, and because theoretical and simulation analyses cannot comprehensively and accurately cover the coupling and mutual influence among practical multi-channel NMR resonators, we further carried out multi-channel automatic tuning-and-matching training and testing on the outer coil of an actual tri-resonance NMR probe. This coil contains two channels, ^13^C and ^15^N, with resonance frequencies of 150.9 MHz and 60.8 MHz, respectively. The circuit topology is shown in Figure 6. The two channels share one detection coil, while their tuning-and-matching circuits are separated and coupled through isolation filtering networks. Thus, the tuning-and-matching performance is affected by the isolation network. On the other hand, there is an inner coil between the outer coil and the detection area, so the outer coil is significantly affected by the coupling of the inner coil. As with the previous ^1^H channel of the inner coil, channel-specific learning and testing were carried out for both channels. During training, the sampling counts of the tuning and matching capacitors for both channels were fixed at 100, and the number of load impedance states was set to 20. After training, we test the minimum reflection value of $S_{11}$ and its offset from the target frequency on different samples, with the target frequencies set to 150.9 MHz for the ^13^C channel and 60.8 MHz for the ^15^N channel. The results in Figure 12 show behavior consistent with that observed on the ^1^H channel.

#### 4.3.4. Comparison Among the Proposed Method, Native CNP, and BP Neural Network

**Figure 13:**
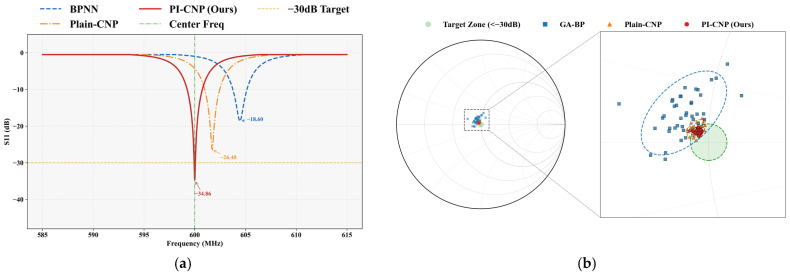
The results of the comparative experiment: (**a**) Comparison of S_11_ responses of BPNN, Plain-CNP, and the proposed PI-CNP on the ^1^H channel; (**b**) Smith chart mapping of the test results on the ^1^H channel.

To verify the performance advantages of the proposed method, we compared it with native CNP and BP neural network. The three methods were trained and evaluated under the same settings, where the number of tuning-capacitor and matching-capacitor sampling points was fixed at 100, and the number of load impedance states was fixed at 20. After training, the test results are shown in Figure 13a, while Figure 13b depicts the corresponding impedance transformations on the Smith chart. The experimental results show that the proposed method achieves substantially better matching accuracy and robustness than the BPNN baseline. Specifically, the point cloud produced by the BP neural network is the most scattered and shows a clear directional spread, indicating not only large errors but also systematic bias, i.e., a persistent deviation in a particular direction away from the matching region. By contrast, the point cloud generated by the proposed method forms a compact cluster concentrated near the center of the Smith chart, corresponding to the 50 Ω matching point. This strong concentration demonstrates the low variance and excellent repeatability of the proposed method, without the spiral oscillation around the chart center or entrapment in local minima often seen in conventional gradient-based search algorithms, such as imaginary-part matching accompanied by real-part mismatch. For the broadband ^1^H channel, the algorithm still suppresses the in-band reflection coefficient to −34.86 dB, whereas the minimum reflection obtained by the BPNN is only about −18.60 dB and therefore fails to satisfy the commonly used engineering threshold of approximately −30 dB. It is also worth noting that the bandwidth characteristics of the measured curve in subsequent real-machine experiments agree well with the simulation prediction, indicating that the model successfully captures the Q-factor characteristics of the real probe coil under load through meta-learning. In contrast, the BPNN exhibits a much larger S_11_ and a more significant offset of the dip frequency relative to the center frequency, indicating that its predicted capacitor combination is more likely to drive the system into an insufficiently matched state. This degradation mainly arises because standard neural networks attempt to fit the global average mapping of the entire parameter space using fixed weights and lacks task-specific contextual adaptation. By contrast, the proposed method uses the encoder to extract a latent representation of the current load from a small number of support-set samples, thereby more stably pulling the matching point back toward the target frequency and significantly reducing the minimum reflection.

### 4.4. Real-Machine Experimental Validation of Automatic Tuning and Matching for NMR Probes

**Figure 14:**
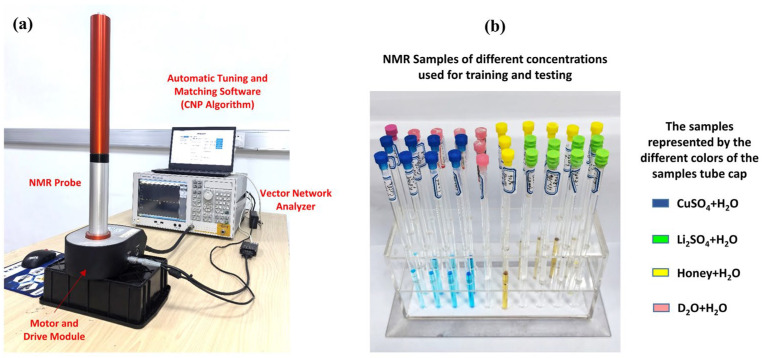
Test system and samples for automatic tuning and matching of an NMR probe. (**a**) Test system. (**b**) NMR samples.

To validate the proposed method on a practical NMR resonator, we constructed an independent automatic tuning-and-matching test platform based on a 600 MHz NMR spectrometer (model 600 X/H, FS; Wuhan Zhongke-Niujin Spectrum Technology Co., Ltd., Wuhan, China). The platform consists of a vector network analyzer (E5071C; Agilent Technologies, Santa Clara, CA, USA), the 600 MHz NMR probe, host computer software implementing the algorithm, motors, and drive modules, thereby forming a closed-loop control system. The motors and driver modules are mounted at the bottom of the probe. The host computer communicates with the driver modules through USB-CAN and controls the motor adjustments. The physical setup is shown in Figure 14. The NMR resonator inside the 600 MHz probe is an inner–outer dual-resonator structure. The inner resonator adopts a saddle coil configuration and serves as the heteronuclear coil with a frequency range of 50–280 MHz for nuclei such as ^13^C, ^31^P, and ^15^N. The outer resonator adopts a field-constrained coil structure with frequency range of 550–610 MHz for nuclei such as ^1^H and ^19^F. The tuning-and-matching circuits of both resonators use improved L-type matching networks and include isolation and filtering circuits. The tuning-and-matching capacitors of each resonator are mechanically linked to stepper motors through rods. The host computer software runs the auto-tuning algorithm, sends the predicted parameters to the stepper motor control modules, and drives the motors to adjust the variable capacitors. The RF input port of the tuning-and-matching module is connected to the vector network analyzer to measure the reflection coefficient S_11_ and the input impedance of the resonator.

During both training and testing, all samples were loaded into standard 5 mm NMR tubes with a volume of approximately 600 μL. The samples included ordinary samples of different concentrations, deuterium oxide (D_2_O + H_2_O) of different concentrations, high-viscosity samples of different concentrations (Honey + H_2_O), ionic copper-sulfate solutions of different concentrations (CuSO_4_ + H_2_O), and lithium sulfate solutions of different concentrations (Li_2_SO_4_ + H_2_O). The concentrations of these samples are shown in Table 4.

The resonance frequency of the ^1^H channel is 600 MHz, while the resonance frequencies of the X-channel nuclei are 60.8 MHz for ^15^N and 150.9 MHz for ^13^C. The tunable ranges of the tuning and matching capacitors on the ^1^H channel are 1–10 pF, while on the X channel, the tunable ranges for each nuclide frequency are 1–35 pF. The stopping criteria of the algorithm were set as |S_11_| < 0.1, corresponding to a return loss of about −20 dB, and the offset distance of the operating frequency $∥f_{S11}-f_{0}∥\;<10\;\mathrm{kHz}$. The loss coefficients λ and β were set to 0.25 and 0.30, respectively. The total number of capacitor sampling points was 100.

In the simulations, an S_11_ threshold of −30 dB was adopted in order to impose a stricter performance criterion on the algorithm and to reserve sufficient accuracy margin for subsequent hardware validation. In the experimental system, achieving this level would require much finer adjustment of the tunable capacitors, which places very stringent demands on the mechanical precision of the stepper motors and the noise immunity of the driving circuitry. On the other hand, when S_11_ reaches −20 dB, the reflected power is only 1%, which is already fully acceptable for practical measurements. Therefore, in the hardware experiments, the S_11_ acceptance criterion was set to −20 dB.

**Figure 15:**
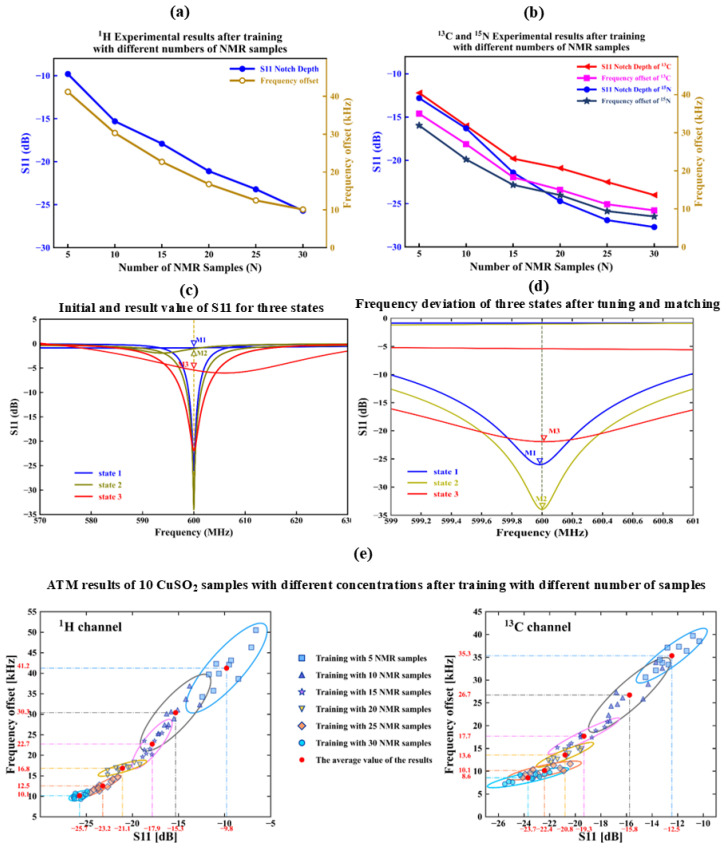
Experimental results of automatic tuning and matching. In (**e**), the ovals indicate the distribution ranges of the test results for each training-sample group, and the red dots represent the average values of the corresponding results.

Using the constructed automatic tuning system and algorithm software, we tested the proposed method in terms of sample requirements, multi-channel performance, and tuning-and-matching accuracy. Six groups of NMR samples were used for training, with group sizes of 5, 10, 15, 20, 25, and 30 samples, respectively. Each group contained evenly distributed concentrations of D_2_O, honey, and lithium ion solution. The concentration of each NMR sample was uniformly spaced from 0.1 mol/L to 3 mol/L. After training, real measurements were performed using ten samples of different concentrations from 0.1 mol/L to 1.2 mol/L and random initial capacitor settings, thereby generating different input impedances. The average tuning-and-matching results over the ten test samples were reported. Figure 15a presents the tuning-and-matching results on the ^1^H channel after learning from 5 to 30 NMR samples. The measured curves show trends consistent with the simulation results: S_11_ improves from −9.7 dB to −25.9 dB, while the frequency offset decreases from 39.1 kHz to 10.3 kHz. With 20 NMR samples, S_11_ reaches −21.9 dB and the frequency offset is 14.8 kHz. Figure 15b shows the multi-channel test results for ^13^C and ^15^N with different sample counts. The measured results are again consistent with simulation. For ^13^C, S_11_ improves from −12.1 dB to −23.8 dB, and the frequency offset decreases from 35.3 kHz to 9.6 kHz. For ^15^N, S_11_ improves from −12.6 dB to −27.5 dB, and the frequency offset decreases from 30.2 kHz to 8.5 kHz. With 20 NMR samples, S_11_ values of −20.4 dB and −24.2 dB are obtained for ^13^C and ^15^N, respectively, while the corresponding frequency offsets are 11.7 kHz and 10.5 kHz. The measured results in Figure 15 demonstrate that, under the proposed method, a fast and accurate automatic tuning network can be established using only a small number of NMR samples. The detailed discrepancies between the measured and simulated curves mainly stem from unknown couplings and parasitic effects included in the learning process of the real NMR probe system, as well as the fact that the NMR samples used for training differ from those used for testing. Figure 15c shows the automatic tuning results of the ^1^H resonator under three severely detuned and mismatched initial states. In state 1, represented by the blue curve, the initial condition is so severely detuned and mismatched that no reflection-coefficient peak appears within the search frequency range. In that case, a conventional search-based algorithm would have to traverse from the minimum capacitor value, greatly increasing the tuning time. By contrast, the proposed method can still produce a good result through neural network inference alone. State 3 corresponds to a sample loaded coil with relatively low Q factor and a very broad initial S_11_ peak, and the proposed method still provides satisfactory tuning results for this case. Figure 15d shows the final deviations from 600 MHz after tuning for the three states, which are −14.5 kHz, +3.2 kHz, and +12.7 kHz, respectively. These frequency offsets are fully acceptable in practical experiments. The real measured results for the three initial states demonstrate that the proposed method has strong sample adaptability and high tuning-and-matching accuracy. The proposed method directly maps the coupling effect between coils and the multi-channel filtering and isolation circuits within coils to the neural network structure and parameters through training, thus greatly overcoming the difficulty of accurately testing the impact of coupling filtering.

During both training and testing, all measured data in the hardware experiments—including input impedance, S_11_, and frequency-offset measurements—were acquired ten times, and the average of the best five measurements was used. The fluctuations in measured S_11_ and frequency offset for ten samples entering and leaving the test setup under different training groups are shown in Figure 15e. As illustrated in the Figure, with the increase in the number of training samples in each group, the fluctuations of S_11_ and frequency offset among the ten test samples within the same group gradually decrease. In the hardware experiments, for the training groups containing 5 and 10 samples, some test results exceeded the acceptance criterion, and occasionally large-deviation failure cases were also observed. These deviations and failures are attributed to the limited number of training samples, which led to insufficient model accuracy.

## 5. Discussion

### 5.1. Computational Resources and Training Time

Because no standard training set exists and the measurement process is affected by sample state and environmental conditions, automatic tuning and matching for NMR must be trained using locally acquired data. As a result, training and testing must be performed on a local workstation. However, the primary role of this workstation is to control the NMR instrument and process spectra. From the standpoint of cost, the local workstation cannot allocate abundant computing resources to the auto-tuning task. Therefore, computational resource consumption and training time are important considerations for neural network-based automatic tuning and matching. The proposed method utilizes a single NVIDIA GeForce RTX 4060 GPU as the accelerating computing unit on a standard workstation, and the training time for a dataset containing 10,000 total capacitor sampling points and 20 NMR samples remains 2 h, which is an acceptable level for initial instrument installation.

### 5.2. Network Architecture and Parameters

Compared with conventional neural network methods, the proposed method can substantially reduce the number of required training samples and achieves good performance on most test samples. However, for the occasional large deviation between the resonance frequency and the target frequency in a single experiment, in addition to the insufficient number of NMR samples used for training, the limited number of network layers and structure also contribute to this deviation. Increasing the number of layers would markedly increase computational cost and impose heavier burden on the available hardware resources. In routine experimental tests, we therefore append a search algorithm after the neural network prediction stage to perform local refinement, further reduce the frequency error, and move the optimal S_11_ point onto the operating frequency. The samples and measurements optimized during these subsequent normal experiments can then be added back into the training set as new sampling points, allowing the network to adaptively refine itself through continual retraining.

### 5.3. Influence of NMR Sample States

Under few-shot training with only a small number of NMR samples, the choice of training sample states is critically important. As shown in Figure 15a, even when the same number of five NMR samples is used for training, the selected samples should in principle cover the full range from the unloaded condition to severely deviated states. However, for highly deviated high-concentration ionic samples, different solvents can have markedly different effects on tuning and matching. For example, high-concentration Li samples and highly concentrated copper-sulfate samples affect the tuning-and-matching behavior differently. We model such samples as equivalent LCR series–parallel loads, and different LC series–parallel forms of these loads lead to different training outcomes. For different coils and channels in a dual-coil structure, different highly ionic NMR samples can likewise induce substantial differences in tuning and matching. Therefore, the selection of NMR samples for few-shot training remains an important issue that deserves further investigation. On the other hand, a more comprehensive set of extended application experiments is still needed to fully demonstrate the adaptability and generalization capability of the proposed method. Future work will therefore evaluate the method using a wider range of sample systems and concentration groups to further examine its sample-adaptation ability, employ different types of probes to validate its generalization performance, and conduct in situ NMR experiments to analyze its adaptive capability for real-time ATM. These directions will be investigated in our subsequent studies.

### 5.4. Generalization Capability

For probe configurations with completely unseen coil structures, tuning-and-matching circuits, shielding structures, or stepper-motor drive mechanisms that are not included in training, the circuit relationship between the input impedance and the coil load may differ substantially. In such cases, the model needs to be retrained to accommodate the resulting changes in circuit behavior, mechanical characteristics, and coupling effects. By contrast, for different probe types that are represented in the training set, the proposed method can generalize across these probe types within a unified model. The generalization discussed in this work therefore refers to task generalization within the family of probe configurations covered during training, rather than zero-shot transfer to completely unseen hardware designs. Under this setting, the proposed CNP-based framework can share task-level priors across different topologies and resonance frequencies and rapidly adapt to new tasks within the same configuration family using only a small number of context measurements. This suggests that the method possesses practically meaningful generalization capability for probe configurations that are structurally related to the training tasks and lie within a similar operating distribution.

### 5.5. Required Runtime

Each capacitor adjustment in NMR automatic tuning and matching mainly consists of four steps: (1) measuring the input impedance and computing the reflection coefficient S_11_; (2) using the current tuning/matching capacitor values together with S_11_ to invoke the automatic tuning-and-matching algorithm and compute the updated capacitor values; (3) driving the motors according to the algorithm-generated capacitor values to adjust the actual capacitances; and (4) measuring the input impedance again and computing S_11_ to check the convergence criterion. If the convergence criterion is not satisfied, these four steps are repeated until convergence is achieved.

In the host computer software developed in this work, time-monitoring checkpoints were set for each step. In the first step, a frequency sweep over the bandwidth is required to obtain the input impedance and S_11_ at each frequency point. In the constructed test system, the time required for the VNA to complete the frequency sweep, acquire the input impedance, and upload the data to the host computer software is 0.85 s. In the second step, the proposed method requires 0.13 s to compute the adjusted capacitor values. In the third step, the time required for the motors to adjust the actual capacitances depends on the initial capacitor positions and is typically 1–5 s. The fourth step requires approximately the same time as the first step. Since the proposed method requires only one capacitor adjustment to reach the desired result, the total required time is approximately 3–7 s. By contrast, existing search-based automatic tuning methods usually require multiple capacitor adjustments before reaching the target result, and their total runtime is typically several times longer than that of the proposed method, usually exceeding 10 s. Therefore, compared with existing automatic tuning-and-matching methods, the proposed method achieves rapid tuning and matching. For certain in situ experiments, where sample-state changes cause tuning-and-matching drift, the proposed method can provide rapid adjustment and thereby achieve a real-time in situ effect.

## 6. Conclusions

This paper proposes a physics-informed few-shot learning method for automatic tuning and matching of NMR resonators over wideband multi-frequency scenarios. The method uses a conditional neural process (CNP) as the core predictive model and formulates the tuning-and-matching process of an NMR RF probe as a function-regression problem conditioned on circuit structure and operating frequency. By learning shared priors over a distribution of tasks, the model can rapidly infer the optimal configuration of tunable components under different loading conditions. Building on this framework, a physics-informed regularization term based on the local sensitivity of the reflection coefficient S_11_ is introduced to constrain network training. This regularization term characterizes the sensitivity of matching degradation to different tunable elements near resonance and enforces stronger penalties on prediction errors along critical directions under high-Q narrowband operating conditions, thereby reducing the physical mismatch risk caused by relying only on mean squared error in parameter space. We further conduct simulation analyses of the model parameters, number of samples, and efficiency, compare the proposed method with a BP neural network baseline, and construct a real automatic tuning-and-matching experimental system for NMR probes. Real-probe validation is performed under different capacitor sampling points, different numbers of NMR samples, multiple circuit topologies, and wideband multi-target tuning scenarios for different resonance nuclei. Compared with conventional neural network methods used in other automatic tuning-and-matching systems and applications, the combination of CNP and physics-informed regularization achieves results that are closer to the desired target on NMR resonators. More importantly, accurate automatic tuning and matching across different circuit topologies and multiple resonance nuclei can be achieved with only a small number of NMR samples. The results show that, owing to the synergy between conditional modeling of practical topological structures and multi-coil coupling on the one hand and physical constraints associated with different NMR sample states on the other hand, the proposed method can, with only 100 tuning-and-matching capacitor sampling points and 20 NMR samples per task, realize unified modeling and high accuracy prediction across multiple circuit topologies, multiple resonance frequency scenarios, and different initial loading states. The method demonstrates strong cross-structure and cross-frequency generalization as well as practical applicability. It also yields consistent improvements in key engineering indicators such as tuning efficiency and the total number of real measurements required per task, especially in cross-topology and cross-frequency settings, and thus provides an efficient and promising solution for emerging NMR technologies and applications such as rapid in situ real-time detection and automatic tracking of tuning and matching.

## Figures and Tables

**Table 1:** Frobenius norms of the Hessian matrix and the relative contribution of off-diagonal terms for L-, Π-, and T-type matching networks under representative load impedances.

Load Impedance	Topology	$∥H_{diag}{∥}_{F}$	$∥H_{off}{∥}_{F}$	ρ
20 + j40 Ω	L	0.07325	0.00502	0.0685
20 + j40 Ω	Π	0.07337	0.00603	0.0821
20 + j40 Ω	T	0.01511	0.00195	0.1291
30 + j60 Ω	L	0.17497	0.03025	0.1728
30 + j60 Ω	Π	0.16717	0.02411	0.1442
30 + j60 Ω	T	0.03583	0.00746	0.2081
40 + j80 Ω	L	0.29896	0.02504	0.0837
40 + j80 Ω	Π	0.27441	0.02610	0.0951
40 + j80 Ω	T	0.04087	0.00442	0.1081

**Table 2:** Equivalent-circuit parameters and tunable-capacitance ranges of different probe channels under no-load conditions.

Channel	Freq. (MHz)	Coil Pos.	Resistance Range $R_{coil}$ (Ω)	Inductance $L_{coil}$ (nH)	Capacitance $C_{par}$ (pF)	Tuning Range (pF)	Matching Range (pF)
^1^H	600	Inner	0.3~6.0	50	0.6	1.0~12.0	1.0~12.0
^13^C	150	Outer	0.2~1.5	120	1.0	2.0~30.0	2.0~30.0
^15^N	60	Outer	0.1~0.8	220	1.2	5.0~100.0	5.0~100.0
^2^H	~92	Inner	0.2~2.0	80	1.0	2.0~30.0	2.0~30.0

**Table 3:** Hyperparameter configurations of the proposed method, the BPNN baseline, and the general training settings.

Category	Parameter	Value
**General**	Activation Function	ReLU
	Weight Decay	1.0 × 10^−3^
**Ours**	Encoder Architecture	MLP: [d_x_→192→192→192→192→96]
	Aggregator Operation	Mean
	Latent Dimension (r)	96
	Decoder Architecture	MLP: [(d_x_ + r)→192→192→192→192→d_y_]
	Optimizer	Adam
	Learning Rate	3.0 × 10^−4^
	Batch Size	48
**BPNN**	**Hyperparameter Search Space:**	
	Optimizer	{SGD, Momentum, Adam}
	Hidden Layers	1~8
	Neurons per Layer	1~256
	Learning Rate	5.0 × 10^−5^~10^−3^
	Dropout Rate	0~0.5

**Table 4:** The concentrations of the samples used for training and testing.

Sample	Concentration							
	Sample 1	Sample 2	Sample 3	Sample 4	Sample 5	Sample 6	Sample 7	Sample 8
CuSO_4_	0.1 mol/L	0.4 mol/L	0.6 mol/L	0.8 mol/L	1.0 mol/L	1.2 mol/L	1.4 mol/L	1.5 mol/L
Li_2_SO_4_	0.1 mol/L	0.5 mol/L	0.8 mol/L	1.0 mol/L	1.3 mol/L	1.6 mol/L	1.8 mol/L	2.0 mol/L
Honey	10%	20%	40%	60%	80%	90%	100%	—
D_2_O	0.1 mol/L	0.5 mol/L	0.9 mol/L	1.3 mol/L	1.6 mol/L	1.8 mol/L	2.0 mol/L	—

## Data Availability

The original contributions presented in this study are included in the article. Further inquiries can be directed to the corresponding author.
